# Peruvian Bathing

**Published:** 1854-07

**Authors:** 


PERUVIAN BATHING.—I took a stroll along the beach, and was much amused at witnessing the singular mode adopted by the ladies for the enjoyment of a water excursion. The bathing-men are Indians, very stout and robust; who, being divested of every species of covering except a pair of drawers, take to the water, each carrying a lady upon his shoulders. The men strike out to swim, and do so without inconveniencing the ladies, who float horizontally on the surface of the water. In this way they are carried a mile or more, and appear to enjoy this novel mode of locomotion extremely.—*Bonelli's Travels in Bolivia.*

